# Screening for Intimate Partner Violence Experience and Use in the Veterans Health Administration

**DOI:** 10.1001/jamanetworkopen.2023.37685

**Published:** 2023-10-13

**Authors:** Galina A. Portnoy, Mark R. Relyea, Candice Presseau, Skye A. Orazietti, LeAnn E. Bruce, Cynthia A. Brandt, Steve Martino

**Affiliations:** 1 VA Connecticut Healthcare System, West Haven; 2 Yale School of Medicine, New Haven, Connecticut; 3 Veterans Healthcare Administration Intimate Partner Violence Assistance Program, Washington, District of Columbia; 4 Western Kentucky University School of Social Work, Bowling Green

## Abstract

**Importance:**

The practice of screening women for intimate partner violence (IPV) in health care settings has been a critical part of responding to this major public health problem. Yet, IPV prevention would be enhanced with detection efforts that extend beyond screening for IPV experiences to identifying those who use violence in relationships as well.

**Objective:**

To determine rates of IPV experiences and use (ie, among perpetrators of IPV) and factors associated with disclosures among adult patients seeking mental health services at the Veterans Health Administration.

**Design, Setting, and Participants:**

This cross-sectional study used electronic medical record data drawn from a quality improvement initiative at 5 Veterans Health Administration medical centers conducted between November 2021 and February 2022 to examine IPV disclosures following concurrent screening for IPV experience and use. Participants included patients engaged in mental health services. Data were analyzed in April and May 2023.

**Exposure:**

Mental health clinicians were trained to screen for IPV experience and use concurrently and instructed to screen all patients encountered through routine mental health care visits during a 3-month period.

**Main Outcomes and Measures:**

Outcomes of interest were past-year prevalence of IPV use and experience, sociodemographic characteristics, and clinical diagnoses among screened patients.

**Results:**

A total of 200 patients were offered IPV screening. Of 155 participants (mean [SD] age, 52.45 [15.65] years; 124 [80.0%] men) with completed screenings, 74 (47.7%) denied past-year IPV experience and use, 76 (49.0%) endorsed past-year IPV experience, and 72 (46.4%) endorsed past-year IPV use, including 67 participants (43.2%) who reported IPV experience and use concurrently; only 9 participants (5.8%) endorsed unidirectional IPV experiences and 5 participants (3.2%) endorsed unidirectional IPV use. Patients who reported past-year IPV experience and use were younger than those who denied IPV (experience: mean difference, −7.34 [95% CI, 2.51-12.17] years; use: mean difference, −7.20 [95% CI, 2.40-12.00] years). Patients with a posttraumatic stress disorder diagnosis were more likely to report IPV use (43 patients [59.7%]) than those without a posttraumatic stress disorder diagnosis (29 patients [40.3%]; odds ratio, 2.14; [95% CI, 1.12-4.06]). No other demographic characteristics or clinical diagnoses were associated with IPV use or experience.

**Conclusions and Relevance:**

In this cross-sectional study of IPV rates and associated factors, screening for IPV found high rates of both IPV experience and use among patients receiving mental health care. These findings highlight the benefit of screening for IPV experience and use concurrently across gender and age. Additionally, the associations found between PTSD and IPV use underscore the importance of strengthening and developing additional targeted treatment for IPV.

## Key Points

**Question:**

What are the rates of intimate partner violence (IPV) experience, IPV use, and factors associated with IPV disclosures among adults presenting for mental health care at 5 Veterans Health Administration medical centers?

**Findings:**

Among 155 patients screened, 43.2% reported both IPV experience and use concurrently, with only 5.8% endorsing unidirectional IPV experiences and 3.2% endorsing unidirectional IPV use. Younger age and posttraumatic stress disorder diagnosis were associated with IPV disclosures.

**Meaning:**

These findings suggest that implementation of screening for IPV experience and use concurrently across genders and ages may present an opportunity to identify and respond to a high-risk population within the health care system.

## Teaser

This cross-sectional study assesses rates of IPV experiences and use and factors associated with disclosures among adults receiving mental health services from the Veterans Health Administration.

## Introduction

The health care system plays a critical public health role in detecting and responding to intimate partner violence (IPV; ie, physical, sexual, and psychological aggression against a current or former intimate partner).^1,2^ Several health care agencies, including the US Preventative Services Task Force, have issued recommendations that clinicians routinely screen women of reproductive age for IPV experience and connect them with follow-up care.^3^ Although screening women for IPV experiences during health care visits is essential to supporting those who have experienced violence, it is insufficient as a primary prevention model of IPV. IPV prevention must extend to detecting those who use violence in relationships and connecting them to appropriate services. Clinicians can serve a key function in identifying IPV use (ie, perpetration), providing referrals, following patients over time,^4,5,6^ and treating IPV and comorbid conditions.

As the largest comprehensive and integrated health care system in the United States, the Veterans Health Administration (VHA) is ideally positioned to screen patients for IPV use and to respond to disclosure with referrals to follow-up care and evidence-based intervention for IPV use.^7^ Historically, efforts to implement screening for IPV experiences have targeted women in primary care settings. Although screening implementation has improved steadily,^8^ logistical, educational, and structural barriers to screening adoption remain.^9,10,11,12^ These primary care barriers, coupled with interdisciplinary clinician beliefs that mental health clinicians may be more effective at assessing IPV use and responding to positive disclosures,^13^ suggest mental health may be a more appropriate setting for initial IPV use screening implementation.

Moreover, although more than 1 in 4 men (28.5%) report experiencing rape, physical violence, or stalking by an intimate partner in their lifetime,^14^ most prior IPV screening research and implementation initiatives have targeted women (particularly of reproductive age). Because minimal screening efforts have focused on the male patient population,^15^ much less is known about IPV screening and disclosures among men.^6^ Implementing screening for IPV experience and use concurrently among patients of all genders presents opportunities for a nongendered, indicated, and comprehensive health care response to IPV. In fact, the Family Violence Prevention Fund developed pilot guidelines for addressing IPV experience and use among men in the health care system based on literature and expert opinion.^6^ Additionally, both clinicians and patients report a preference for IPV screening that includes the detection of IPV use and experience concurrently in the VHA,^13^ and studies have consistently identified bidirectional IPV (ie, both partners experiencing and using violence in the relationship) as a common pattern of violence among veterans and service members.^16,17^ However, little research examining IPV experience and use in the same sample exists, limiting our understanding of needs, health care utilization, and outcomes among patients who experience and use violence concurrently. The goal of this observational study was to determine rates of IPV experience and use and factors associated with IPV disclosure following concurrent IPV experience and use screening among patients engaged in VHA mental health services.

### Methods

The data for this cross-sectional study were drawn from a quality improvement (QI) initiative and received a determination of nonresearch by the VA Connecticut Healthcare system’s institutional review board. As a clinical operations initiative, participant consent was not required. Only deidentified data were used for analyses. The study followed the Strengthening the Reporting of Observational Studies in Epidemiology (STROBE) reporting guideline.

### Enrollment and Participants

A total of 23 clinicians across 5 VHA health care facilities electively enrolled to participate in an IPV screening implementation QI initiative. Sites with clinical capacity for responding to positive IPV experience and use screening results, approval of local leadership, and at least 5 interested clinicians were selected. Participating clinicians were independently licensed, had supervisor permission, and provided clinical services to at least 10 unique patients in a typical week. The 5 participating VA health care facilities were diverse in geography (1 in the Northeast, 3 in the Midwest, and 1 in the South), size (ranging from approximately 43 000 to 97 000 unique patients during fiscal year 2022), and VA’s coding of facility complexity (3 were highest complexity, 1 high complexity, and 1 moderate complexity).^18^ Patients with at least 1 mental health clinical encounter during the 90-day period were eligible for screening. Clinicians were instructed to screen all patients for IPV experience and use concurrently 1 time during routine mental health care appointments across a 3-month period, including both in-person and virtual visits.

Of 1720 patients with clinical encounters eligible for screening, a total of 200 patients across 5 VHA medical centers were offered screening for IPV experience and use. Of 200 patients offered screening, 45 (22.5%) were not screened due to 30 (66.6%) failing environmental safety checks and 15 (33.3%) declining consent for screening. Of the 30 patients who reported unsafe to screen cases, 1 (3.3%) had a child older than 2 years present, 21 (70.0%) had an adult other than the patient present, and 8 (26.7%) failed environmental checks for an unspecified reason.

### Clinical Tools and Data Sources

Patients were screened using the 10-item Relationship Violence Use and Experience Screener (RVUES), which includes the 5-item Extended-Hurt/Insult/Threaten/Scream (E-HITS) tool to screen for IPV experiences^19,20,21^ and the 5-item Modified E-HITS tool to screen for IPV use,^22^ with each behavior queried simultaneously (eg, “you screamed or cursed at your partner” followed by “your partner screamed or cursed at you”) alongside behaviorally specific examples, per recommendations for assessing potentially stigmatizing or stressful events.^23^ Consistent with the original E-HITS, responses of past-year IPV experience and use were reported on 5-point scales ranging from 0 (never or not in the past 12 months, but it has happened before) to 4 (frequently). Per VHA policy for positive IPV screening results, responses of 1 or greater on any item resulted in a positive result (ie, any past-year IPV reported). The full measure is available in eTable 1 in Supplement 1.

Using data extracted from the VHA Corporate Data Warehouse,^24^ we identified patients who were administered the RVUES during a documented mental health visit at the VHA medical centers enrolled in the QI initiative during a 90-day screening window between November 2021 and February 2022. In addition to the data variables associated with the IPV screener, we extracted sociodemographic characteristics (age, sex, race, ethnicity, marital status, and housing status) collected through patient self-report and relevant clinical diagnoses (military sexual trauma [MST], alcohol use disorders, sleep disorders, depressive disorders, anxiety disorders, and posttraumatic stress disorder [PTSD]) via VHA health factors and mental and physical health conditions. Race was categorized as Black or African American, White, and other races, including American Indian or Alaska Native, Asian, Native Hawaiian or Other Pacific Islander, or those who selected more than 1 race, and ethnicity was categorized as Hispanic or Latino and not Hispanic or Latino. We included race and ethnicity, along with other sociodemographic characteristics, to assess potential associations with screening outcomes. Clinical diagnoses were identified using *International Classification of Diseases, Ninth Revision* (*ICD-9*) and *International Statistical Classification of Diseases and Related Health Problems, Tenth Revision *(*ICD-10*) codes. Health factors are standardized structured data in clinical notes recorded in the electronic health record that enable information to be maintained and queried. For all clinical diagnoses, we coded patients as having a positive result if the *ICD-9* or *ICD-10* codes were present in either 2 outpatient visits or 1 inpatient within 1 year prior to the patient’s index visit, ie, the first visit in the facility’s 90-day screening window (eTable 2 in Supplement 1).

### Statistical Analysis

We calculated frequencies to determine rates of IPV experience and use among patients who were screened for IPV. For patients with positive results, we calculated the range, mean, SD, and median for each IPV screener item to examine the frequency of each IPV disclosure. We used *t* tests and logistic regression to assess bivariate associations of positive disclosures of IPV with continuous variables (ie, age) using mean differences and categorical variables (ie, other sociodemographic characteristics and clinical diagnoses) using odds ratios. We set the statistical significance level at *P* < .05 and used 2-tailed tests. Nearly all variables had full data. We used pairwise deletion for 3 variables with missing data (marital status, 2 patients [1.3%]; MST, 2 patients [1.3%]; and race, 10 patients [6.4%]). All analyses were conducted in R statistical software version 4.1.3 (R Project for Statistical Computing) using arsenal version 3.6.3 and lsr version 0.5.2 packages. Data were analyzed in April and May 2023.

## Results

The final sample included 155 patients who completed screening. The mean (SD) age was 52.45 (15.65) years (range, 22-91 years). The sample included 124 (80.0%) men and 31 (20.0%) women and 93 unmarried patients (60.0%), 60 married patients (38.7%), and 2 patients (1.3%) with unknown marital status. By race, 20 patients (12.9%) were Black or African American, 123 patients (79.4%) were White, 2 patients (1.2%) reported other races, and 10 patients (6.5%) had missing race data; 32 patients (20.6%) were Hispanic. Most patients were sufficiently housed (117 patients [75.5%]), with 38 patients (24.5%) experiencing homelessness or marginally housed. Approximately half of the screenings (74 screenings [47.7%]) occurred in person; 52 screenings (33.5%) were conducted via video, and 29 screenings (18.7%) were conducted via telephone.

In terms of past-year IPV, 74 patients (47.7%) denied IPV experience and use, 76 patients (49.0%) endorsed IPV experience, and 72 patients (46.5%) endorsed IPV use (Table 1). There was significant overlap among patients who reported IPV experience and use, such that 67 patients (43.2%) reported both, with only 9 patients (5.8%) endorsing unidirectional IPV experiences and 5 patients (3.2%) endorsing unidirectional IPV use. Most patients reported moderate psychological IPV (ie, screamed or cursed and insulted or talked down: 76 patients [49.0%] reported experience; 72 patients [46.5%] reported use), with fewer reporting severe psychological violence (threatened: 20 patients [12.9%] reported experience; 16 patients [10.3%] reported use), physical violence (physically hurt: 13 patients [8.4%] reported experience; 6 patients [3.9%] reported use), or sexual violence (forced or pressured sexual contact: 6 patients [3.9%] reported experience; 1 patient [0.6%] reported use). Among those who endorsed IPV, median IPV experience responses were never (threats, physical violence, sexual violence) and sometimes (screamed at or insulted) and median IPV use responses were never (threats, physical violence, sexual violence), rarely (insulted), and sometimes (screamed at or insulted).

**Table 1.** Rates of Past-Year IPV Experience and Use

IPV measure	Patients reporting IPV, No. (%) (N = 155)	Item-level descriptive statistics among patients reporting IPV^a^	
		Median (range)	Mean (SD)
Experience			
Any	76 (49.0)	NA	NA
Scream	71 (45.8)	2 (0-4)	2.04 (1.17)
Insult	57 (36.8)	2 (0-4)	1.80 (1.43)
Threaten	20 (12.9)	0 (0-4)	0.53 (1.04)
Physical IPV	13 (8.4)	0 (0-3)	0.33 (0.79)
Sexual IPV	6 (3.9)	0 (0-4)	0.14 (0.60)
Use			
Any	72 (46.5)	NA	NA
Scream	69 (44.5)	2 (0-4)	1.94 (1.11)
Insult	45 (29.0)	1 (0-4)	1.06 (1.09)
Threaten	16 (10.3)	0 (0-4)	0.39 (0.83)
Physical IPV	6 (3.9)	0 (0-2)	0.14 (0.48)
Sexual IPV	1 (0.6)	0 (0-1)	0.01 (0.12)

Abbreviations: IPV, intimate partner violence; NA, not applicable.

^a^ Item response options for past-year IPV were coded as 0, indicating never or not in the past 12 months, but it has happened before; 1, rarely, including just once; 2, sometimes; 3, often; and 4, frequently.

Age was the only sociodemographic characteristic associated with positive IPV disclosures. Patients who reported both past-year IPV experience and use were younger than those who did not (experience: mean difference, −7.34 [95% CI, 2.51-12.17] years; use: mean difference, −7.20 [95% CI, 2.40-12.00] years) (Table 2), with mean (SD) ages of 48.71 (13.77) years for participants reporting IPV experience and 48.60 (13.68) for those reporting IPV use. Patients with a PTSD diagnosis were more likely to report past-year IPV use (43 patients [55.8%]) compared with those without PTSD (29 patients [37.2%]; odds ratio, 2.14; [95% CI, 1.12-4.06]) (Table 3). No other mental or physical health conditions were associated with IPV experience or use disclosures.

**Table 2.** Associations of Demographics and Past-Year IPV Experience and Use

Characteristic	Total patients, No. (%) (N = 155)	IPV experience			IPV use		
		Patients, No. (%)		OR (95% CI)	Patients, No. (%)		OR (95% CI)
		No	Yes		No	Yes	
Age, mean (SD), y	52.45 (15.65)	56.05 (16.57)	48.71 (13.77)	7.34 (2.51, 12.17)^a^	55.80 (16.54)	48.60 (13.68)	7.20 (2.40, 12.00)^a^
Gender							
Women	31 (20.0)	18 (22.8)	13 (17.1)	1 [Reference]	17 (20.5)	14 (19.4)	1 [Reference]
Men	124 (80.0)	61 (77.2)	63 (82.9)	1.43 (0.65-3.17)	66 (79.5)	58 (80.6)	1.07 (0.48-2.35)
Marital status							
Unmarried	93 (60.0)	52 (65.8)	41 (55.4)	1 [Reference]	55 (66.3)	38 (54.3)	1 [Reference]
Married	60 (38.7)	27 (34.2)	33 (44.6)	1.55 (0.81-2.98)	28 (33.7)	32 (45.7)	1.65 (0.86-3.18)
Unknown	2 (1.3)	NA	NA	NA	NA	NA	NA
Race^b^							
Black or African American	20 (12.9)	11 (15.1)	9 (12.9)	0.83 (0.32-2.15)	10 (13.2)	10 (14.9)	1.16 (0.45-2.98)
White	123 (79.4)	62 (84.9)	61 (87.1)	1 [Reference]	66 (86.8)	57 (85.1)	1 [Reference]
Other races	2 (1.3)	NA	NA	NA	NA	NA	NA
Unknown	10 (6.5)	NA	NA	NA	NA	NA	NA
Ethnicity							
Hispanic	32 (20.6)	20 (25.3)	12 (15.8)	0.55 (0.25-1.23)	22 (26.5)	10 (13.9)	0.45 (0.20-1.02)
Non-Hispanic	123 (79.4)	59 (74.7)	64 (84.2)	1 [Reference]	61 (73.5)	62 (86.1)	1 [Reference]
Housing status							
Sufficiently housed	117 (75.5)	63 (79.7)	54 (71.1)	1 [Reference]	67 (80.7)	50 (69.4)	1 [Reference]
Homeless or marginally housed	38 (24.5)	16 (20.3)	22 (28.9)	1.60 (0.77-3.36)	16 (19.3)	22 (30.6)	1.84 (0.88-3.87)

Abbreviations: IPV, intimate partner violence; NA, not applicable; OR, odds ratio.

^a^ Expressed as mean difference (95% CI).

^b^ Statistical tests for race only included comparisons between Black or African American and White veterans due to small samples sizes of other groups. The other races category included those who identified as American Indian or Alaska Native, Asian, Native Hawaiian or Other Pacific Islander, or those who selected more than 1 race.

**Table 3.** Associations of Clinical Diagnoses and Past-Year IPV Experience and Use

Diagnosis	Total patients, No. (%)	IPV experience			IPV use		
		Patients, No. (%)		OR (95% CI)	Patients, No. (%)		OR (95% CI)
		No	Yes		No	Yes	
Alcohol use							
No	93 (60.0)	45 (57.0)	48 (63.2)	1 [Reference]	47 (56.6)	46 (63.9)	1 [Reference]
Yes	62 (40.0)	34 (43.0)	28 (36.8)	0.77 (0.41-1.47)	36 (43.4)	26 (36.1)	0.74 (0.39-1.41)
Anxiety							
No	102 (65.8)	55 (69.6)	47 (61.8)	1 [Reference]	57 (68.7)	45 (62.5)	1 [Reference]
Yes	53 (34.2)	24 (30.4)	29 (38.2)	1.41 (0.73-2.75)	26 (31.3)	27 (37.5)	1.32 (0.68-2.56)
Depression							
No	80 (51.6)	41 (51.9)	39 (51.3)	1 [Reference]	44 (53.0)	36 (50.0)	1 [Reference]
Yes	75 (48.4)	38 (48.1)	37 (48.7)	1.02 (0.55-1.92)	39 (47.0)	36 (50.0)	1.13 (0.60-2.12)
MST							
No	125 (80.6)	60 (77.9)	65 (85.5)	1 [Reference]	65 (80.2)	60 (83.3)	1 [Reference]
Yes	28 (18.1)	17 (22.1)	11 (14.5)	0.60 (0.26-1.38)	16 (19.8)	12 (16.7)	0.81 (0.36-1.86)
Unknown	2 (1.3)	NA	NA	NA	NA	NA	NA
PTSD							
No	78 (50.3)	43 (54.4)	35 (46.1)	1 [Reference]	49 (59.0)	29 (40.3)	1 [Reference]
Yes	77 (49.7)	36 (45.6)	41 (53.9)	1.40 (0.74-2.63)	34 (41.0)	43 (59.7)	2.14 (1.12-4.06)
Sleep disorder							
No	114 (73.5)	59 (74.7)	55 (72.4)	1 [Reference]	62 (74.7)	52 (72.2)	1 [Reference]
Yes	41 (26.5)	20 (25.3)	21 (27.6)	1.13 (0.55-2.30)	21 (25.3)	20 (27.8)	1.14 (0.56-2.32)

Abbreviations: IPV, intimate partner violence; MST, military sexual trauma; NA, not applicable; OR, odds ratio; PTSD, posttraumatic stress disorder.

## Discussion

This cross-sectional study examined screening for IPV experience and use concurrently and factors associated with IPV disclosures among patients presenting for mental health care at 5 VHA medical centers. Over the last decade, screening for IPV experience among women has increased steadily in health care, including the VHA.^8^ However, to identify and prevent IPV, initiatives are needed to detect patients who use violence in relationships in addition to those who experience it. A major strength of this study was use of an IPV screening tool that assessed IPV experience and use simultaneously, allowing for more comprehensive appraisal of violence in patients’ relationships. The fact that most patients in our sample were men is also noteworthy, as very few IPV screening initiatives have targeted the male patient population. Additionally, including patients beyond reproductive age is important, as adults older than 45 years are an underrepresented group in the IPV screening literature.

Despite a relatively small proportion of eligible patients offered screening, the screening rate in this pilot reflects prior rates of IPV screening adoption at VHA.^8^ In addition to building on literature identifying barriers to implementing screening for IPV experiences among women of reproductive age,^8,10,25,26,27,28^ further research, training, and implementation efforts are needed to enhance clinician adoption of IPV screening, including for patients of all genders and ages, as well as for IPV experiences and use concurrently. In terms of representativeness, the subsample of patients offered screening in this sample did not differ demographically or diagnostically from the 1520 patients not offered screening. Moreover, the 1720 eligible patients were similar to patients presenting for mental health care at the VHA in terms of age, race, marital status, PTSD, and depressive disorder.^29,30^ The eligible patients included higher rates of Hispanic patients (15.8%) and women (15.99%) compared with VHA mental health samples (approximately 5% Hispanic patients and approximately 12% women patients),^29,30^ with neither characteristic associated with IPV disclosures. These populations are important to include, as women are the fastest growing veteran demographic^31^ and Hispanic veterans are the fastest growing racial and ethnic group at the VHA.^32^

Screening for IPV experience and use concurrently revealed high rates of both IPV types. Half of the patients screened reported experiencing past-year IPV, a finding consistent with studies demonstrating similar rates of IPV experience in mental health treatment seeking populations.^33^ Although less is known regarding screening for IPV use in health care settings, our finding that almost half of patients disclosed past-year IPV use aligns with IPV use rates found in treatment seeking research samples.^34,35,36^

There were no gender differences in IPV experience or use disclosures. Moreover, most patients who reported IPV reported experience and use concurrently (ie, bidirectional IPV), with minimal unidirectional IPV present in the sample (5.8% unidirectional IPV experience and 3.2% unidirectional IPV use). These findings underscore the need to move beyond a gender-targeted violence prevention model that perpetuates ideology regarding men’s use and women’s experience of violence toward more comprehensive conceptualization of IPV to include screening all patients for IPV experience and use regardless of gender. However, gender symmetry in IPV experience and use on a brief screening tool does not necessarily equate to equivalence in the function or impact of IPV. Historically, men are more likely to cause serious IPV-related injury compared with women, and women are more likely to experience greater fear and physical harm resulting from IPV.^37,38^ Accordingly, more nuanced research and clinical assessment of IPV with enhanced focus on the impact of violence and its function (eg, context in which it occurs, who initiates it, and motive, such as self-defense) will add meaningful knowledge to the field. Improved understanding of the nature and function of violence in relationships will, in turn, reveal opportunities for more specified, targeted, and appropriate prevention and intervention.

Consistent with meta-analyses across samples demonstrating that psychological IPV is the most common form of IPV, most IPV endorsed in our study was psychological.^39^ This finding has important clinical and public health implications. Psychological IPV is a robust risk factor associated with physical aggression, and identifying psychological IPV levels has distinctive utility for future violence escalation.^40^ Apart from the potential for escalation, psychological IPV itself has significant physical and mental health consequences.^41,42,43^ In a large population-based study, psychological IPV was associated with negative outcomes (eg, depression, poor health), and when considered alongside physical IPV experience, psychological IPV was associated with greater risk of adverse health outcomes than physical IPV.^42^ Clinically, these findings reveal opportunities for intervention development focused on psychological IPV. For example, increasing skills related to cognitive flexibility has been identified as an important target among individuals who have experienced trauma and can help mitigate risk for externalizing behavior, such as aggression and IPV use.^44^

Age was the only sociodemographic characteristic associated with IPV in this study. Our finding that younger patients were more likely to experience and use past-year IPV compared with older patients aligns with past research demonstrating that younger individuals are disproportionately affected by IPV.^3,45,46^ However, the mean age of patients reporting experiencing (48.7 years) and using (48.6 years) IPV in our sample was older than reproductive age, underscoring the benefit of screening middle-aged and older patients. This finding is consistent with emerging evidence demonstrating that IPV remains prevalent and associated with morbidity among women 45 years and older^47^ and brings into question the current recommendation of restricting IPV screening to women of reproductive age.^3^ Additionally, the global population is becoming older and living longer than in previous decades, leading to greater health care demands and utilization. In particular, with the baby boomer generation experiencing higher rates of chronic disease, disability, and poorer self-rated health than prior generations,^48^ more patients from this generation are likely to present within the health care system. The combination of a significant paucity in IPV research among middle-aged and older individuals, increased health care utilization in the aging population, and persistence of IPV experience with associated morbidity across these age groups highlights the importance of screening patients for IPV experience and use concurrently across the lifespan.^45,47^

In this study, PTSD was the only mental or physical health condition significantly associated with IPV disclosures. However, a diagnosis of PTSD was only positively associated with past-year IPV use, not IPV experience, contrary to evidence demonstrating that IPV experiences are associated with PTSD in samples across gender.^49^ The associations of PTSD symptoms with aggression and violence perpetration is well established across veteran, civilian, epidemiological, and clinical samples^50,51,52,53,54,55^ and bidirectional IPV samples.^56^ Our findings emphasize that treatment for IPV use must be both trauma-informed and focused on underlying mechanisms between PTSD and violence perpetration in relationships, such as deficits in social information processing (ie, the cognitive-emotional process for interpreting and responding to external stimuli),^55^ akin to the IPV use intervention implemented in the VHA.^7^ Furthermore, there may be additive prevention benefits to implementing screening and education for IPV use and experience concurrently in health care settings with high levels of health care utilization among patients who use IPV, including clinical services for PTSD, chronic sleep problems, anxiety, depression, chronic pain, and stomach or digestive disorders.^57^

### Limitations

This study has several limitations. First, it is a nonrandomized, retrospective analysis of data collected as part of a QI initiative. Additionally, although we assessed sociodemographic and diagnostic factors, there may be other patient and clinician differences not captured that may confer differential risk for IPV experience or use disclosures (eg, clinicians’ decisions to screen and patients’ willingness to disclose IPV). Additionally, the sample used in this study is relatively small and focused on patients accessing VHA mental health care services, which may limit generalizability to other populations and clinical settings. Furthermore, this initiative was the first time that the validated IPV experience and IPV use brief screeners were used in the form of the integrated RVUES instrument; more research is needed to examine the accuracy and acceptability of such a clinical tool.

## Conclusions

This cross-sectional study found that nearly half of patients presenting for VHA mental health care reported past-year IPV experiences and use. PTSD was associated with IPV use but not IPV experience, underscoring potential distinct treatment targets for patients with PTSD in service of IPV prevention. These findings demonstrate that screening for IPV experience and use concurrently and across genders and ages presents an opportunity to identify and respond to a unique high-risk population within mental health care. In identifying and targeting common cooccurrence of psychological IPV use and experiences, there is potential to mitigate subsequent escalations of violence within intimate partner relationships. This study can help integrated health care systems, like the VHA, plan for broader implementation of concurrent screening for IPV experience and use and support the development of additional targeted treatment for IPV.

## Notes

Supplement 1. **eTable 1.** Relationship Violence Use and Experience Screener (RVUES) **eTable 2.** *International Classification of Diseases* (*ICD*) Diagnosis Codes

Supplement 2.
Data Sharing Statement
