# Supplementary material for: Screening for Intimate Partner Violence Experience and Use in the Veterans Health Administration
Source: JAMA Netw Open. 2023 Oct 13;6(10):e2337685. doi: 10.1001/jamanetworkopen.2023.37685 (PMC10576210; doi:10.1001/jamanetworkopen.2023.37685)
Supplement: Supplement 1. — eTable 1. Relationship Violence Use and Experience Screener (RVUES) eTable 2. International Classification of Diseases (ICD) Diagnosis Codes [file jamanetwopen-e2337685-s001.pdf]

Supplemental Online Content

Portnoy GA, Relyea MR, Presseau C, et al. Screening for intimate partner violence experience and use in the Veterans Health Administration. *JAMA Netw Open*. 2023;6(10):e2337685.
doi:10.1001/jamanetworkopen.2023.37685

eTable 1. Relationship Violence Use and Experience Screener (RVUES)

eTable 2. *International Classification of Diseases* (*ICD*) Diagnosis Codes

This supplemental material has been provided by the authors to give readers additional information about their work.

© 2023 Portnoy GA et al. *JAMA Network Open.*

eTable 1. Relationship Violence Use and Experience Screener (RVUES)

No matter how well a couple gets along, there are times when they disagree, get annoyed with the other person, want different things from each other, or just have spats or fights because they are in a bad mood, are tired, or for some other reason. Couples also have many different ways of trying to settle their differences. This is a list of things that might happen when you have differences.

Please select how often you did each of these things to a past or current partner in the last 12 months, and how often a past or current partner did them to you in the last 12 months. Examples of partners can include a spouse, boyfriend or girlfriend, someone you dated, or someone with whom you had a sexual relationship.

**How often has this happened in the last 12 months?**	**Never**	**Rarely (including just once)**	**Sometimes**	**Often**	**Frequently**
**You screamed or cursed at your partner**<br>*Examples: yelled at them, swore at them, etc.*	0	1	2	3	4
**Your partner screamed or cursed at you**<br>*Examples: yelled at you, swore at you, etc.*	0	1	2	3	4
**You insulted or talked down to your partner**<br>*Examples: called them names, belittled them, etc.*	0	1	2	3	4
**Your partner insulted or talked down to you**<br>*Examples: called you names, belittled you, etc.*	0	1	2	3	4
**You threatened your partner with harm**<br>*Examples: threatened to hit, throw something, or hurt them; intimidated them; punched a wall in front of them, etc.*	0	1	2	3	4
**Your partner threatened you with harm**<br>*Examples: threatened to hit, throw something, or hurt you, intimidated you; punched a wall in front of you, etc.*	0	1	2	3	4
**You physically hurt your partner**<br>*Examples: pushed, slapped, grabbed, punched, kicked, etc.*	0	1	2	3	4
**Your partner physically hurt you**<br>*Examples: pushed, slapped, grabbed, punched, kicked, etc.*	0	1	2	3	4
**You forced or pressured your partner to have sexual contact against their will or when they were unable to say no**<br>*Examples: pressured them or insisted on sexual activities when they didn't want to, insisted on sex without a condom, engaged in sexual activities when they were asleep or too drunk to give permission, used force or threat of force, etc.*	0	1	2	3	4
**Your partner forced or pressured you to have sexual contact against your will or when you were unable to say no**<br>*Examples: pressured you or insisted on sexual activities when you didn't want to, insisted on sex without a condom, engaged in sexual activities with you when you were asleep or too drunk to give permission, used force or threat of force, etc.*	0	1	2	3	4

© 2023 Portnoy GA et al. *JAMA Network Open.*

eTable 2. *International Classification of Diseases* (*ICD*) Diagnosis Codes

This supplemental material is provided for additional information regarding ICD codes used to identify potential comorbidities in the corresponding study.

**Comorbidity**	**Diagnoses included**	**Unique ICD 9 and 10 Codes**
Alcohol use disorders	• Alcohol Use Disorder<br>• Alcohol Abuse or Dependence, Alcohol Psychosis<br>• Alcohol Related Disorders	303.00, 303.01, 303.02, 303.03, 303.90, 303.91, 303.92, 303.93, 305.00, 305.01, 305.02, 305.03, F10.10, F10.11, F10.120, F10.121, F10.129, F10.14, F10.150, F10.151, F10.159, F10.180, F10.181, F10.182, F10.188, F10.19, F10.20, F10.21, F10.220, F10.221, F10.229, F10.230, F10.231, F10.232, F10.239, F10.24, F10.250, F10.251, F10.259, F10.26, F10.27, F10.280, F10.281, F10.282, F10.288, F10.29, 291.0, 291.1, 291.2, 291.3, 291.4, 291.5, 291.8, 291.81, 291.82, 291.89, 291.9, 357.5, 425.5, 535.30, 535.31, 571.0, 571.1, 571.2, 571.3, 790.3, 980.0, 980.8, 980.9, E860.0, E860.9, F10.920, F10.921, F10.929, F10.94, F10.950, F10.951, F10.959, F10.96, F10.97, F10.980, F10.981, F10.982, F10.988, F10.99, G62.1, I42.6, K29.20, K29.21, K70.0, K70.10, K70.11, K70.2, K70.30, K70.31, K70.40, K70.41, K70.9, V11.3
Sleep disorders	• Nightmares<br>• Parasomnia<br>• Obstructive Sleep Apnea<br>• Other Disorders of Sleep<br>• Insomnia<br>• Sleep Apnea	307.46, 307.47, F51.4, F51.5, 307.46, 327.4, 327.40, 327.41, 327.42, 327.43, 327.44, 327.49, G47.50, G47.51, G47.52, G47.53, G47.54, G47.59, 327.23, G47.33, 307.40, 307.41, 307.42, 307.48, 307.49, 327.8, F51.2, F51.3, F51.8, F51.9, 327.20, 327.21, 327.22, 327.29, G47.30, G47.31, G47.32, G47.39, 327.01, 780.52, F51.01, F51.02, F51.03, F51.09, G47.00, G47.01, V69.4, Z72.820
Depressive disorders	• Major Depressive Disorder<br>• Other Depression	296.20, 296.21, 296.22, 296.23, 296.24, 296.25, 296.26, 296.30, 296.31, 296.32, 296.33, 296.34, 296.35, 296.36, F32.0, F32.1, F32.2, F32.3, F32.4, F32.5, F32.9, F33.0, F33.1, F33.2, F33.3, F33.40, F33.41, F33.42, F33.9, 309.81, F43.10, F43.11, F43.12, 293.83, 296.90, 296.99, 298.0, 300.4, 301.11, 301.12, 301.13, 309.0, 309.1, 311., 311.0, 311.9, F06.31, F06.32, F32.8, F32.81, F32.89, F33.8, F34.1
Anxiety disorders	• Anxiety state, unspecified*<br>• General anxiety disorder*	300.00, F06.4, F40.00, F40.01, F40.02, F40.10, F40.11, F40.210, F40.218, F40.220, F40.228, F40.230, F40.231, F40.232, F40.233, F40.240, F40.241, F40.242, F40.243, F40.248, F40.290, F40.291, F40.298, F40.8, F40.9, F41.0, F41.3, F41.8, F41.9, F42., F42.2, F42.3, F42.4, F42.8, F42.9, F45.20, F45.21, F45.29, 300.02, F41.1
Posttraumatic stress disorder	• Posttraumatic Stress Disorder	309.81, F43.10, F43.11, F43.12

*Per VA's Program Evaluation and Resource Center (PERC) definition

© 2023 Portnoy GA et al. *JAMA Network Open.*
