# Supplementary material for: Screening for Intimate Partner Violence Experience and Use in the Veterans Health Administration
Source: JAMA Netw Open. 2023 Oct 13;6(10):e2337685. doi: 10.1001/jamanetworkopen.2023.37685 (PMC10576210; doi:10.1001/jamanetworkopen.2023.37685)
Supplement: Supplement 2. — Data Sharing Statement [file jamanetwopen-e2337685-s002.pdf]

Data Sharing Statement

Portnoy. Screening for Intimate Partner Violence Experience and Use in the Veterans Health Administration. *JAMA Netw Open*. Published October 13, 2023.
doi:10.1001/jamanetworkopen.2023.37685

Data
Data available: Yes
Data types: Deidentified participant data, Data dictionary
How to access data: Those interested in the deidentified dataset and data dictionary underlying this manuscript can send an email request to the corresponding author: galina.portnoy@va.gov
When available: With publication

Supporting Documents
Document types: Other (please specify)
Additional Information: Supplemental material has been provided by the authors for additional information about the International Classification of Disease (ICD-9 and 10) diagnosis codes used to identify potential comorbidities.
How to access documents: galina.portnoy@va.gov
When available: With publication

Additional Information
Who can access the data: Anyone
Types of analyses: For a specified purpose
Mechanisms of data availability: With a signed data access agreement
Any additional restrictions: Data sharing will be required to comply with government procedures
